# Distant metastasis time to event analysis with CNNs in independent head and neck cancer cohorts

**DOI:** 10.1038/s41598-021-85671-y

**Published:** 2021-03-19

**Authors:** Elia Lombardo, Christopher Kurz, Sebastian Marschner, Michele Avanzo, Vito Gagliardi, Giuseppe Fanetti, Giovanni Franchin, Joseph Stancanello, Stefanie Corradini, Maximilian Niyazi, Claus Belka, Katia Parodi, Marco Riboldi, Guillaume Landry

**Affiliations:** 1 Department of Radiation Oncology, University Hospital, LMU Munich, Munich, 81377 Germany; 2 Department of Medical Physics, Faculty of Physics, Ludwig-Maximilians-Universität München, Garching, 85748 Germany; 3 German Cancer Consortium (DKTK), Munich, 81377 Germany; 4 Medical Physics Department, Centro di Riferimento Oncologico di Aviano (CRO) IRCCS, 33081 Aviano, Italy; 5 Radiation Oncology Department, Centro di Riferimento Oncologico di Aviano (CRO) IRCCS, 33081 Aviano, Italy; 6 Guerbet SA, Villepinte, France

**Keywords:** Cancer, Biomarkers, Medical research, Oncology

## Abstract

Deep learning models based on medical images play an increasingly important role for cancer outcome prediction. The standard approach involves usage of convolutional neural networks (CNNs) to automatically extract relevant features from the patient’s image and perform a binary classification of the occurrence of a given clinical endpoint. In this work, a 2D-CNN and a 3D-CNN for the binary classification of distant metastasis (DM) occurrence in head and neck cancer patients were extended to perform time-to-event analysis. The newly built CNNs incorporate censoring information and output DM-free probability curves as a function of time for every patient. In total, 1037 patients were used to build and assess the performance of the time-to-event model. Training and validation was based on 294 patients also used in a previous benchmark classification study while for testing 743 patients from three independent cohorts were used. The best network could reproduce the good results from 3-fold cross validation [Harrell’s concordance indices (HCIs) of 0.78, 0.74 and 0.80] in two out of three testing cohorts (HCIs of 0.88, 0.67 and 0.77). Additionally, the capability of the models for patient stratification into high and low-risk groups was investigated, the CNNs being able to significantly stratify all three testing cohorts. Results suggest that image-based deep learning models show good reliability for DM time-to-event analysis and could be used for treatment personalisation.

## Introduction

Biology-driven personalised treatment is a landmark in the development of precision radiation oncology. Over the past years, several biomarkers (e.g. human papilloma virus (HPV) status or positron emission tomography based hypoxia levels) have been proposed to help clinical decision making for improved management of certain cancers^1^.

Radiomics relies on non-invasive biomarkers based on advanced imaging analytics^2^ and has been shown to be able to unravel tumor phenotype in multiple studies^3–6^. The typical radiomics workflow involves imaging of the patient, identification of the gross tumor volume (GTV), conversion of images to higher dimensional data (i.e. radiomic features) and the subsequent integration and mining of these data for model building. Thus, radiomics allows to build diagnostic, prognostic and predictive models for clinical outcomes using imaging data which is acquired as a part of clinical routine^7^. However, several challenges have to be faced during radiomic signature development such as issues in reproducibility, standardisation in both the image acquisition, the handcrafted feature extraction and the statistical model building, and other limitations and pitfalls^8^. For instance, Welch et al.^9^ have shown that a previously developed set of radiomic features was a surrogate for tumor volume, highlighting the need for simple baseline models to be compared with advanced radiomic signatures.

Conventional radiomic models are built using machine learning algorithms: for binary classification tasks (e.g. whether the patient survives or not) typically random forests, support vector machines or artificial neural networks (ANNs) are used, while for survival analysis (i.e. predicting time-to-events or risks for clinical outcomes) Cox proportional hazards regression, random survival forests, and support vector survival methods are commonly used^10^. In the past few years, a sub-field of machine learning called deep learning^11^ has been widely and successfully adopted in a variety of fields. For medical applications, convolutional neural networks (CNNs) have been extensively used as they take spatial information into account and are therefore the preferred architecture for image-related tasks^12^. Compared to traditional radiomics, deep learning based radiomics approaches exploit the inherent non-linearity of deep neural networks to learn relevant features automatically^13^. This enables end-to-end analysis as the contoured or cropped images are given to the algorithm which outputs directly the predictions, therefore skipping the step of handcrafted feature extraction and the efforts connected with it.

Deep learning based models have been proven successful in a wide range of medical applications including classification of skin cancer^14^ or extranodal extensions in head and neck squamous cell carcinoma^15^, fully-automated localisation and segmentation of rectal cancer^16^ and mortality risk stratification for lung cancer patients^17^ to name a few. Within radiology and radiotherapy, examples are detection of mammographic lesions^18^, cone-beam computed tomography (CT) intensity correction^19^ or synthetic CT generation from magnetic resonance images^20,21^. Also they have been shown to either equal or outperform their engineered features counterparts in many classification tasks. For instance Diamant et al.^22^ could show that a CNN trained from scratch on pre-treatment CT images of head and neck cancer patients outperformed a traditional radiomics model developed in a previous study^5^ using exactly the same CT images. In both studies distant metastasis (DM), loco-regional failure and overall survival were used as endpoints for 300 patients available on the cancer imaging archive (TCIA)^23,24^ and coming from four different hospitals in Quebec, Canada. Compared to dichotomised classification, only few deep learning based survival analysis models have been applied to the field of medical imaging, although several models have already been proposed^25–27^. Katzman et al.^26^ implemented DeepSurv, a deep neural network which outputs a single number, that is, the log-risk function of a Cox proportional hazards model allowing for personalized treatment recommendations. Gensheimer et al.^27^ proposed Nnet-survival, a scalable discrete-time survival model for neural networks capable of outputting survival curves for every patient in a given time span.

Head and neck cancers are a set of very heterogeneous malignancies which are diagnosed worldwide more than 830,000 times and lead to more than 430,000 deaths every year^28^. Prognosis of these cancers depends on several factors including tumour site, TNM-stage, extracapsular nodal extension and HPV status, with 5-year survival rates that have been shown to vary from 90% for HPV$^{-}$ early-stage tumors, to 80–87% for HPV$^{+}$ and 37–58% for HPV$^{-}$ tumours presenting cervical lymph node metastasis^29^. Therefore, models that are able to identify high and low risk patients and that can support clinical decision making are highly desirable and could for instance be used for dose de-escalation in radiotherapy, with the potential of decreasing long-term toxicity.

**Figure 1:**
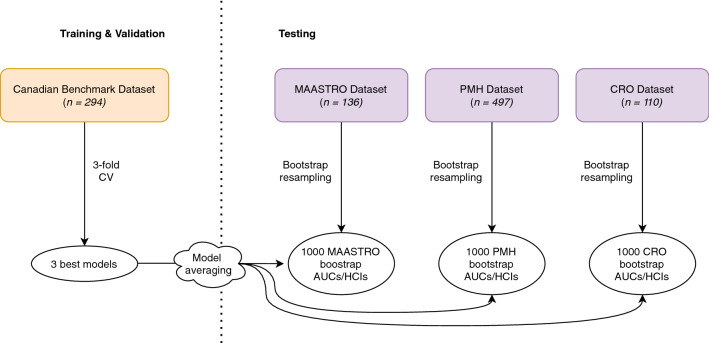
Cohorts training, validation and testing subdivision and analysis workflow. Firstly, the Canadian dataset used by Diamant et al.^22^ was used for 3-fold CV to find the hyper-parameters leading to the 3 best validation models. Then, the best validation models were applied to 1000 bootstrap samples of each of the 3 independent testing sets. The predictions of the 3 validation models for each bootstrap sample were averaged to obtain one model averaged prediction per bootstrap replicate. Prediction performance on one cohort was evaluated in terms of area under the curve (AUC) for binary classification and Harrell’s concordance index (HCI) for time-to-event analysis.

With this work we wanted to assess the ability of CNN based approaches to accurately predict the occurrence of DM on several independent testing cohorts. To do so, we first downloaded the same 300 patients available on TCIA^23^ and replicated the results obtained by Diamant et al. on this cohort using cross validation (CV) on 294 patients with a 2D-CNN. Additionally, we performed CV with a 3D-CNN, in order to exploit the full volumetric information of the tumour. Then, we downloaded two additional head and neck cancer cohorts from TCIA, 136 patients from MAASTRO clinic in the Netherlands^30^ and 497 patients from Princess Margaret Cancer Centre (PMH) in Canada^31^ and obtained an additional cohort of 110 patients from Centro di Riferimento Oncologico (CRO) in Italy as a third independent testing cohort. This allowed us to perform a retrospective multicentric study with 1037 CTs of different head and neck cancer patients in total. An overview of the workflow and the cohort subdivision for training, validation and testing is shown in Figure 1.

Additionally, we extended the task of the network from binary classification to time-to-event analysis as no CNN being able to predict DM risk was found in literature. By combining Diamant’s network in 2D and 3D with the survival model by Gensheimer et al.^27^ we have therefore constructed a deep learning framework which incorporates censoring information and is capable to output in an end-to-end fashion DM-free probability curves as function of time for every patient given the contoured pre-treatment CT image of the corresponding patient. Throughout this study, we also compared the performance of the CNNs with the performance of an ANN based on multiple clinical variables such as overall TNM stage, tumor site and volume to assess whether the CNN outperforms more classical prognostic models when it comes to discriminative performance or patient stratification. We also combined the image based CNNs with the clinical covariates based ANN in what we call CNN+Clinical, to see if a combined input could improve overall performance. Finally, to gain a better understanding of the importance of texture for the CNN prediction we performed a binary masking experiment with the input GTV and compared the performance on the testing sets with the performance achieved when using the standard image input.

## Results

### Benchmark study comparison

To verify that a CNN similar to the one built in the benchmark study^22^ is able to transfer its predictive performance to independent testing sets we first had to replicate the results obtained for the validation. For this, we built a 2D-CNN as well as 3D-CNN for classification and performed 3-fold CV on the same patient cohort used in the benchmark study. In general, the area under the curves (AUCs) obtained with the CNNs were smaller or in line with the ones obtained by Diamant et al. (see Table 1). We also performed 3-fold CV with an ANN based on clinical variables and obtained CV AUCs which were higher than for our 2D-CNN and comparable to the ones of the 3D-CNN. For the combined CNN+Clinical models, we achieved validation AUCs which were on average higher than for the CNNs alone.

**Table 1:** Comparison of AUCs for CV in Diamant’s study^22^ and CV and testing in our study.

Model	CV diamant^22^	3-fold CV	Test MAASTRO	Test PMH	Test CRO
2D-CNN	0.80–0.88	0.75; 0.83; 0.67	0.81 (0.73–0.89)	0.62 (0.57–0.67)	0.80 (0.68–0.89)
2D-CNN+Clinical	–	0.86; 0.74; 0.80	0.89 (0.83–0.94)	0.66 (0.61–0.71)	0.71 (0.56–0.83)
3D-CNN	–	0.83; 0.77; 0.79	0.82 (0.74–0.90)	0.63 (0.58–0.68)	0.65 (0.47–0.82)
3D-CNN+Clinical	–	0.82; 0.80; 0.90	0.87 (0.80–0.94)	0.66 (0.61–0.71)	0.67 (0.55–0.79)
ANN	–	0.83; 0.73; 0.86	0.86 (0.79–0.92)	0.66 (0.61–0.71)	0.74 (0.62–0.84)

The numbers shown for the 3-fold CV are the AUCs obtained on the CV subsets. Numbers for the testing sets represent the median AUC and in brackets the 83% confidence intervals obtained via bootstrap re-sampling. Note that the AUCs in columns 2 and 3 can be compared directly as the CV was performed on the same cohort^23^.

### Adding independent testing cohorts

Without changing any hyper-parameter we applied the three best models from validation on a large number of patients never seen by the networks and coming from different institutions than the train and validation cohort, i.e. on three independent testing sets (Fig. 1). To be able to statistically compare results among different cohorts we performed bootstrap re-sampling on all the testing cohorts. The median AUC with 83% confidence interval resulting from bootstrapping (see "Statistical analysis" subsection under "Methods" for the reason behind using 83% confidence) is given for each testing cohort in Table 1. The model which most consistently transferred good validation into independent testing was the 2D-CNN, being able to achieve good AUCs of around 0.8 for two out of three testing cohorts, the PMH cohort being statistically worse than the other two, with a median AUC of 0.62. The 3D-CNN, the 2D and 3D CNN+Clinical and the ANN achieved very good results on the MAASTRO cohort, the other two cohorts being worse. In general, models were always able to transfer good validation prediction performance to the MAASTRO testing cohort, while for the CRO cohort the performance depended on the architecture. For the PMH cohort no network architecture yielded a median AUC above 0.66.

### Performing time-to-event analysis

**Figure 2:**
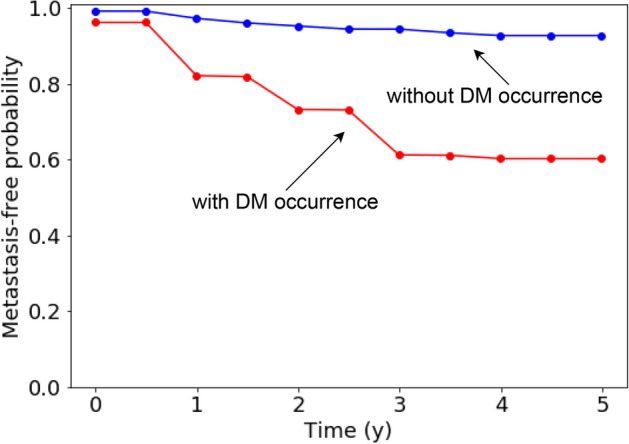
Time-to-event network output. The DM-free probability curves shown are taken from two exemplary patients of the CRO testing cohort using the 3D-CNN as prognostic model. Note that the network output is at pre-defined discrete time points, the lines being drawn for visualisation purposes.

Several authors^25–27^ have underlined the issue of censored data when performing binary outcome classification and have proposed extensions to deep learning models that are able to incorporate time-to-event information and thus censoring. Therefore, we extended our CNNs based on Diamant’s study^22^ with the discrete-time survival model by Gensheimer et al.^27^. The same extension was also applied to the CNN+Clinical and the ANN. These networks were trained from scratch following the same workflow used for binary classification, additionally incorporating the time-to-event/follow-up times for every patient. The output of such networks is for every patient a DM-free probability curve, as shown in Fig. 2 for two selected patients with and without DM occurrence.

We first performed time-to-event analysis using the same image input as for the binary classification task, i.e. the CT masked with the primary and lymph node GTVs. Additionally, to analyse the importance of texture for the CNNs we performed an experiment: instead of giving the network the GTV with re-scaled Hounsfield Units inside (standard image input), we gave the networks a binary image, i.e. the GTV with all values inside set to $+1$ and everything outside the GTV set to $-1$ (see Supplementary Fig. S1). In this way we can exclude that during the learning process the networks are looking at anything else except from the tumor volume and 3D shape (for the 3D-CNN) or the tumor area and 2D shape (for the 2D-CNN). For the binary masking experiment we took the best hyper-parameters obtained with standard image input and then followed again the exact same data splitting and workflow as depicted in Fig. 1, to ensure that no information flows from the testing cohorts into the hyper-parameters used for training.

To evaluate the performance of time-to-event models on different cohorts we used HCI^32^ as it incorporates censoring in the computation of the metric and is therefore well suited to assess performance for this task.

Table 2 shows the results for CV and testing for both the standard image input and the binary masked image input. In general, trends from binary classification have been confirmed for the MAASTRO and the PMH cohort while we observed an overall improvement for the CRO cohort. Both the 2D/3D CNNs and the 2D/3D CNNs+Clinical were able to transfer good validation results to two out of three cohorts. The same holds for the ANN, although results were on average slightly worse than for the CNNs and the CNNs+Clinical. Again, the PMH dataset was found to be worst for all models.

Of interest is also the fact that when performing the binary masking experiment the HCI for the 3D-CNN and for the 3D-CNN+Clinical decreased by no more than 0.03 (i.e. not significantly). A more substantial drop (yet still not significant in terms of confidence intervals) was observed for the 2D-CNN and the 2D-CNN+Clinical.

**Table 2:** Comparison of HCIs for CV and testing for different time-to-event models and the two different image input scenarios.

Model	Image input	3-fold CV	Test MAASTRO	Test PMH	Test CRO
2D-CNN	Standard	0.74; 0.75; 0.71	0.81 (0.70–0.91)	0.64 (0.59–0.69)	0.86 (0.76–0.93)
2D-CNN+Clinical	Standard	0.78; 0.88; 0.80	0.88 (0.82–0.94)	0.67 (0.61–0.71)	0.77 (0.66–0.87)
3D-CNN	Standard	0.78; 0.74; 0.80	0.88 (0.80–0.93)	0.67 (0.62–0.71)	0.77 (0.60–0.90)
3D-CNN+Clinical	Standard	0.82; 0.84; 0.88	0.88 (0.80–0.94)	0.66 (0.61–0.71)	0.74 (0.64–0.85)
2D-CNN	Binary	0.67; 0.76; 0.64	0.75 (0.61–0.86)	0.69 (0.64–0.73)	0.76 (0.63–0.89)
2D-CNN+Clinical	Binary	0.90; 0.74; 0.78	0.87 (0.81–0.93)	0.66 (0.61–0.70)	0.74 (0.64–0.83)
3D-CNN	Binary	0.77; 0.78; 0.80	0.87 (0.80–0.92)	0.67 (0.62–0.72)	0.77 (0.65–0.87)
3D-CNN+Clinical	Binary	0.90; 0.80; 0.77	0.85 (0.75–0.94)	0.67 (0.62–0.72)	0.72 (0.61–0.82)
ANN	–	0.78; 0.82; 0.76	0.87 (0.81–0.92)	0.66 (0.61–0.71)	0.74 (0.62–0.84)

The numbers shown for the 3-fold CV are HCIs obtained for the CV subsets. Numbers for the testing sets represent the median HCI and in brackets the 83% confidence intervals obtained via bootstrap re-sampling. Note that HCIs for the ANN are shown only once because the only input are clinical variables of the corresponding patients.

**Figure 3:**
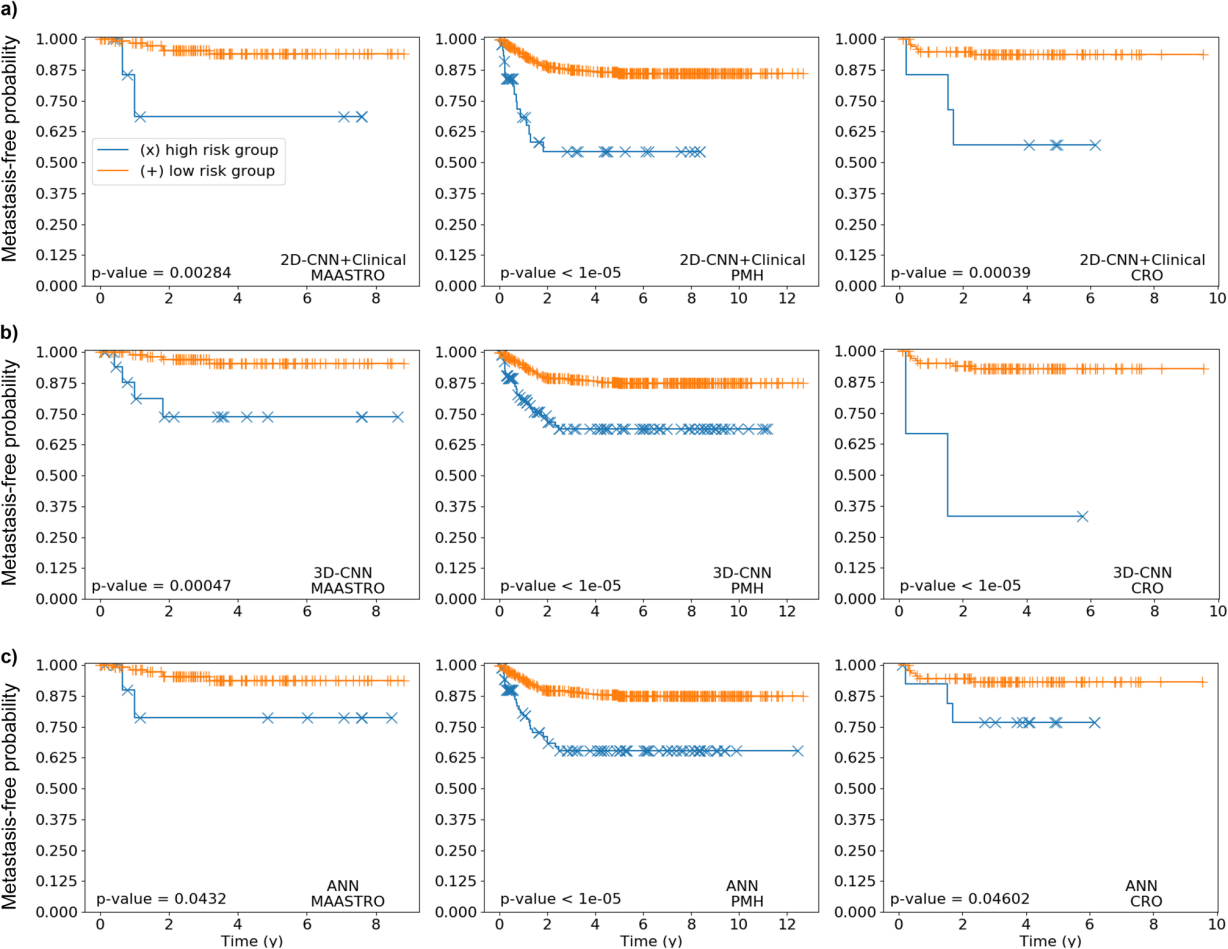
Kaplan–Meier curves obtained when applying time-to-event models on the 3 testing cohorts. The testing patients were stratified into two groups by using an optimised threshold obtained during CV. The displayed p values were computed using the log rank test. (**a**) Kaplan–Meier curves resulting from 2D-CNN+Clinical. (**b**) Kaplan–Meier curves resulting from 3D-CNN. (**c**) Kaplan–Meier curves resulting from ANN based on clinical variables.

In addition to the assessment of the discriminative power according to HCI, we also analysed patient stratification capability of the time-to-event networks. To do so, we found an optimal threshold to split the CV cohorts into high- and low-risk patients and then applied this threshold to the three independent testing cohorts. To infer whether the stratification on the testing cohorts was significant we applied the log rank test on the two patient groups. The resulting p value and corresponding Kaplan–Meier plots for the two groups are shown in Fig. 3 for the 3D-CNN and the 2D-CNN+Clinical with standard image input (because the average performance over all testing cohorts was the best and equal for these two models) and for the ANN as baseline. A complete list containing the patient stratification p values for all tested models can be found as Supplementary Table S1.

As can be seen in Fig. 3a,b, the CNNs were able to significantly stratify all three testing cohorts into high- and low-risk patient subgroups. Even though also the ANN significantly stratified all three testing cohorts (Fig. 3c), it should be noted that, as visible in the plots, the obtained difference between the two risk groups is less pronounced if compared to the 2D CNN+Clinical and the 3D CNN in two (MAASTRO and CRO) out of three testing cohorts.

## Discussion

In general, it was shown that CT based 2D and 3D CNNs are well performing and reliable models for DM time-to-event analysis in head and neck cancer patients. The binary classification 2D-CNN built de novo in the benchmark study by Diamant et al.^22^ was chosen as a starting point, also making use of public data on the TCIA^23,24^. Compared to Diamant et al. we used a 3D network in addition to a 2D network and both the primary and the lymph node GTVs instead of only the primary GTV. When assessing whether this increase in complexity was justified based on the CV cohort, we observed an average increase in performance when going from the 2D-CNN to the 3D-CNN (Table 1, 3rd column). This result suggested that some relevant information for DM outcome prediction is contained in the full 3D volume.

The benchmark study did not evaluate model performance on an independent test set. To infer whether results obtained on the validation set (on which the hyper-parameter tuning is performed) can be transferred to independent testing sets we gathered three additional data sets from three different institutions in North America (PMH) and Europe (MAASTRO, CRO) and applied our trained models to them (Fig. 1). As can be seen in Table 1, only the 2D-CNN was able to transfer good average CV AUCs to two out of three testing sets, contradicting the results obtained for CV alone and thus underlining the importance of using independent testing sets. To analyse whether a simpler model would be able to achieve similar results we also built an ANN based solely on clinical variables and obtained on average similar results to the 2D-CNN, although the values varied more between the different testing cohorts.

To see if combined clinical and image information leads to better performance we extended the 2D and 3D CNNs to include clinical covariates but obtained no improvement in performance when looking at testing cohorts. It should be noted that we only used 7 clinical variables as these were the only ones available in all 4 cohorts. Using more covariates might increase the performance of the ANN and the CNN+Clinical.

To overcome the issue of patients loss during follow-up, we extended all architectures with the time-to-event model by Gensheimer et al.^27^. This allowed for incorporating censoring information in the training process and enabled output of DM-free probability curves at different time points for every patient (see Fig. 2). This could allow for better personalised treatments as the additional information on how fast an event occurs is available (which is particularly relevant for e.g. older patients). When looking at discriminative performance in terms of HCI we found that both the 2D and the 3D CNNs were able to transfer good validation results to two (MAASTRO and CRO) out of three testing cohorts (Table 2), unlike the binary classification CNNs. This suggests that incorporating censoring information may lead to a more consistent performance. The 2D-CNN+Clinical achieved exactly the same testing performance as the 3D-CNN while the average testing HCI of the 2D-CNN, the 3D-CNN+Clinical and the ANN was slightly worse.

Although results were significantly better than 0.5, the PMH cohort was the worst for both tasks, leading at best to an AUC of 0.66 (83% CI 0.61–0.71) and a HCI of 0.69 (83% CI 0.64–0.73). Considering the substantial GTV volume difference between the other cohorts and PMH (see Table 3) we exploited the high number of patients in this cohort (497) to tackle the performance problem by training time-to-event CNNs and ANNs from scratch using only the PMH cohort. The best hyper-parameters were found by applying 3-fold CV several times on the first 50% of the cohort. Again, the three CV models leading to the best validation HCI were then applied on the second 50% of the cohort (using model averaging and bootstrap re-sampling). However, we achieved no improvement on average, the best result being a HCI of 0.69 (see Supplementary Table S2). In a previously published study, Kwan et al.^4^ had also used a subset of PMH with a traditional radiomics model to discriminate DM risk. Their best HCI of 0.71 is in good agreement with our results, hinting towards the fact that no matter which models are used, some cohorts might be more challenging than others, at least in terms of discriminative power.

In this work, we also performed a binary masking experiment on the CNN input image. Instead of giving the network the GTV contours with re-scaled Hounsfield Units inside, we set all values inside the GTV to $+1$ and then fed the resulting image to the time-to-event CNNs (see Supplementary Fig. S1). The performance of the networks did not change significantly for both the 2D-CNN and the 3D-CNN, although a more notable drop was observed for the 2D-CNN (Table 2). These results suggest that while tumor texture might increase performance if the CNN is limited to 2D, it is of less relevance for the 3D-CNN, the volume and 3D shape of the GTVp and GTVn being sufficient to achieve good testing performances. The latter finding is in agreement with the result obtained by Welch et al.^9^ using a traditional radiomics model.

Finally, we also assessed the performance of our models in terms patient stratification capability. To prevent information flow from testing to training/validation we found a threshold to split patients in high- and low-risk groups using the validation cohort and then applied this threshold to the three testing cohorts. Our best performing models in terms of HCI, that is, the 2D-CNN+Clinical and the 3D-CNN with standard image as input, were also found to achieve good results for patient stratification, being able to significantly separate all three testing cohorts (log-rank test p values $< 0.05$). Clinically speaking, this result might be of relevance as CNNs could be used to determine risk groups that might not respond well, or respond better. This information could then be used for treatment (or dose level) adaptation. The ANN was slightly worse than the CNNs, the separation between the high-risk and the low-risk groups being less pronounced for the MAASTRO and CRO cohorts, as can be seen in Fig. 3.

Recently, a study with 291 patients investigating different deep learning approaches for predicting loco-regional head and neck tumor control was published^33^. Censoring information was incorporated in all models using a different time-to-event model than in our study^26^. Similarly to this work, the authors found a CT based time-to-event 3D-CNN to be the best performing model, both in terms of discriminative power as measured with HCI and in terms of patient stratification capability. Regarding the performance of their baseline clinical model in terms of HCI, a more considerable drop with respect to the 3D-CNN was found. In contrast to our study, they found the 2D-CNNs to be substantially worse than the 3D-CNNs, although it should be kept in mind that the prognosis endpoint was a different one.

The main drawback of this study consists in its retrospective nature. Consequently, not all clinical background information was available. For example, for many patients there is uncertainty on whether they received surgery after the primary radiotherapy treatment (see “Patient cohorts” subsection), a fact which might change the outcome of the treatment. For the PMH cohort this information is missing for all patients which might be one of the reasons for its underperformance. Therefore, large prospective studies could be important in the assessment of outcome prediction models. Additionally, as a consequence of our findings it is even more crucial to reduce inter-physician variability in the contouring stage, as the binary masking experiment underlined the importance of tumor volume and shape. Future studies could address whether more advanced CNN architectures like ResNet^34^ or DenseNet^35^ improve DM outcome prediction. It also remains to be seen if additional imaging (e.g. positron emission tomography or magnetic resonance imaging) or genomic biomarkers can enhance the performance of deep learning algorithms for binary classification or time-to-event prediction.

In conclusion, this report highlighted via a large number of independent testing patients the efficacy of image based deep learning models for DM binary classification and time-to-event analysis.

## Methods

### Patient cohorts

For this study, 4 different cohorts totalling 1037 patients with head and neck cancer were used. All patients selected for this study received either radiation alone or chemo-radiation as main treatment and have a follow-up time larger than 2 years. All patients which had a metastasis at time of diagnosis were excluded.

- Canadian benchmark cohort: consists of 298 patients treated at 4 different hospitals in Quebec, Canada. Out of 298 patients, 294 were used for this study as few patients had to be excluded due to data corruption or non-clear identification of the RTSTRUCT corresponding to the CT. This cohort was used in the benchmark study by Diamant et al.^22^ and in a traditional radiomics study^5^ on which the former was based. All patients received either radiation alone or chemo-radiation as main treatment modality. For three out of four hospitals information on surgery was not available. Out of the 88 patients for whom surgery information was available, 10 received it. More detailed information on this cohort can be found on TCIA^23^.
- MAASTRO cohort: consists of 137 patients treated at Maastricht Radiation Oncology clinic in the Netherlands. Out of 137 patients, 1 had to be excluded due to metastasis at first diagnosis. This cohort was used for survival analysis by Aerts et al.^3^ in one of the first traditional radiomics studies, but to our knowledge it was never used to predict DM outcome. All patients were treated with radiotherapy and for all patients information on surgery was available: on 6 out of 136 cancer surgery was performed. More information on this cohort can be found on TCIA^30^.
- PMH cohort: consists of 606 patients treated at Princess Margaret Cancer Centre in Canada. Out of 606, 497 were used for this study as the remaining ones had to be exluded due to short follow-up time, missing RTSTRUCT or missing GTV for instance due to surgical resection. Although information on surgery was not available in the clinical data sheet, contour names for some patients suggested that a surgery or a resection was performed. All patients were treated with either radiotherapy alone or chemo-radiotherapy as primary treatment. Subsets of this cohort were used in several studies^4,9,36^, although only Kwan et al. applied a traditional radiomics model on it to discriminate DM risk. More detailed information on this cohort can be found on TCIA^31^.
- CRO cohort: consist of 110 patients treated at Centro di Riferimento Oncologico Aviano in Italy. Only those patients were selected who were concordant with the other cohorts in terms of clinical specifications (e.g. tumor site, treatment modality, etc.). All patients were treated with either radiation alone or with chemo-radiation as primary treatment and no patient received surgery. This cohort was obtained via a collaboration and can be shared upon request.

For all patients, the pre-treatment CT and primary (GTVp) and lymph node (GTVn) gross tumor volumes contoured by expert physicians of the corresponding hospitals were available. For all cohorts but PMH the GTVns were clearly labeled in the RTSTRUCT. For PMH every region of interest was contoured, thus to select the GTVn we looked into every patient manually and labeled as GTVns those nodes which were inside the clinical target volume. After that, under consultation of an expert physician in our department, we excluded all previously selected lymph nodes which had a volume smaller than 2 cm$^3$. Table 3 compares some relevant clinical variables and specifics among the different cohorts used in this study.

The Canadian benchmark cohort, MAASTRO and PMH are publicly available datasets and were retrieved from TCIA in anonymised form. The CRO patient data was analysed retrospectively, in anonymised form and are part of two studies approved by the Unique Regional Ethics Committee, with following approval numbers: CRO-2017-50 and CRO-2019-66. All methods were carried out in accordance with relevant guidelines and regulations. Informed consent was obtained from all patients.

**Table 3:** Comparison of clinical variables and percentage of patients having a DM among the different cohorts used in this study.

Cohort	Male/female	Median age (years)	Overall stage I/II/III/IV	Median GTVp + GTVn	DM
Benchmark	76/24%	63	1/9/20/68%	31.9 cm$^3$	13.3%
MAASTRO	81/19%	61	17/8/16/57%	19.0 cm$^3$	5.8%
PMH	80/20%	61	1/6/14/78%	39.4 cm$^3$	14.3%
CRO	63/37%	57	20/21/10/46%	23.0 cm$^3$	7.9%

### Network architectures

**Figure 4:**
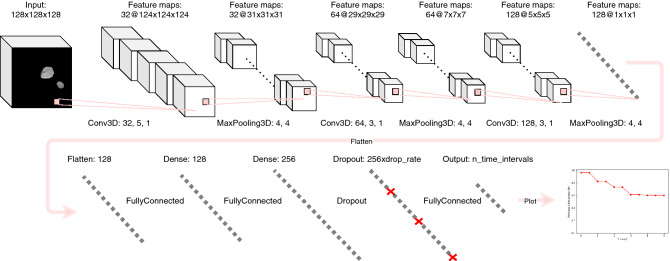
Time-to-event 3D convolutional neural network architecture. The general network architecture is based on the 2D CNN implemented by Diamant et al.^22^ plus the discrete time-to-event model by Gensheimer et al.^27^. Numbers for the convolutional layers represent the number of kernels, the kernel size and the stride. Numbers for the max-pooling layers represent the pool size and the stride.

Both our 2D-CNN and our 3D-CNN architectures are inspired by the 2D-CNN built by Diamant et al.^22^. As depicted in Fig. 4, our 3D-CNN (and 2D-CNN) comprises 3 convolutional blocks followed by two fully connected layers, one dropout layer and the final output layer. Every convolutional block is formed by a convolutional layer, which allows to change the representation of the input data while taking into account spatial information, a max-pooling layer, which introduces invariance of the network to small translations of the input^11^ and drastically reduces the number of parameters, and a parametric rectified linear unit (PReLu) as non-linear activation function (not shown in Fig. 4). After the convolutional blocks, the input image has been translated into a set of features with shape (128, 1, 1, 1) which are flattened to a vector of shape (128, ) which in turn is given as input to the two following fully connected layers (again followed by a PReLu activation function which is not shown in Fig. 4). After that follows a dropout layer, in which a number of neurons given by the *dropout_rate* hyper-parameter is set to zero to prevent the network from over-fitting, and finally the last fully connected layer which, together with a sigmoid activation function (not shown), forms the output of the network For the binary classification network this is a single number between 0 and $+1$.

The main differences of our network with respect to the benchmark study^22^ are that we used both a 3D-CNN and a 2D-CNN and that we extended the CNNs with the survival model by Gensheimer et al.^27^ to be able to incorporate time-to-event information.

To extend the network from 2D to 3D we used 3D Keras built-in functions for the convolutions and the max-pooling instead of the corresponding 2D functions. The image input for our 2D-CNN was the $256 \times 256$ axial slice with the highest number of tumor pixels as in Diamant’s study^22^. The input for the 3D-CNN was the full 3D image of $256 \times 256 \times 256$. To avoid memory issues both the 2D and the 3D input image were reduced to $128 \times 128 (\times 128$) with the random cropping augmentation. To adapt the binary classification CNNs to time-to-event analysis the only change which had to be done at architecture level was to increase the number of neurons in the final layer from one to *n_time_intervals*, i.e. to the number of time intervals for which the network outputs an event probability (see Fig. 4). More details on the implementation of the discrete-time survival model^27^ within our framework can be found in the next subsection.

To compare the performance of the CNN with a simple baseline model based solely on clinical variables we opted for a shallow artificial neural network. To be specific, our ANN has seven clinical variables as input for every patient, that is, patient’s age, gender, tumor site, overall stage, T-stage, N-stage and primary plus lymph node tumor volume. Age in years and total tumor volume in cm$^3$ were divided by 100 and given directly to the ANN while the other five variables, being of categorical nature, were one-hot encoded and then given to the ANN. The input layer of the network was followed by one fully connected hidden layer with 14 neurons, a ReLu activation function, a dropout layer and the final fully connected layer with sigmoid activation function. Exactly as for the CNN, the last layer of the ANN has 1 neuron for the binary classification task and *n_time_intervals* neurons for the time-to-event task.

Finally, in an attempt to get a better performance by combining the two previous models, we also constructed what we call the CNN+Clinical. This network has the same architecture as the CNN but has as an additional input to the masked CT also the same seven clinical variables which were used for the ANN. The clinical variables are given to the CNN+Clinical at the level of the flattening layer by concatenating the 128 features from the convolutional blocks with the vector containing the seven (partly) one-hot encoded clinical covariates. The resulting vector is then fed to the remaining architecture with fully connected layers exactly as for the normal CNN (Fig. 4).

### Time-to-event analysis

Although binary classification models as the ones built in^5,17,22^ achieve good prediction results, they are limited by the fact that time-to-event information is discarded during model training. Especially in some situations, for instance when predicting death or metastasis occurrence for older patients, it is fundamental for treatment customisation to know how fast the event would occur. Additionally, binary classification models usually are trained on a specific time point (e.g. 2-year OS) so they would need to be re-trained to make predictions for a different one^27^.To overcome these issues we extended Diamant’s network to include time-to-event information by incorporating the survival model by Gensheimer et al.^27^. Their discrete-time survival model has been implemented in Keras^37^ (https://github.com/MGensheimer/nnet-survival) and allows to train deep neural networks while taking into account patient follow-up times and outputs discrete event probability/survival curves for each patient. To our knowledge, the only applications of Gensheimer’s model to the field of medical imaging are the study by Zhang et al.^38^, who recently showed that their CT-based CNN survival model was able to outperform a Cox proportional hazards model based on radiomic features for pancreatic ductal adenocarcinoma and the study by Kim et al.^39^, who developed a CT-based deep learning model which successfully predicts disease-free survival for lung adenocarcinoma patients.

To train our time-to-event CNN, the custom loss function *surv_likelihood* provided by the authors is used. At architecture level, the number of neurons in the final connected layer has to be changed in order to match the desired number of output time intervals (Fig. 4). To be specific, every neuron in the final layer outputs the conditional probability that the patient does not get a DM in the corresponding time interval. To obtain a “survival curve” as displayed in Fig. 4 the conditional probabilities up to a certain time interval have to be cumulatively multiplied. For our experiments we used 10 time intervals with a spacing of half a year.

To evaluate the performance by taking into account time-to-event information (conversely to the AUC) we used HCI^32^ which is defined as the fraction of patients for which the predictions and the outcomes are concordant. For our purposes we adapted Lifelines’ concordance index (https://lifelines.readthedocs.io/en/latest/lifelines.utils.html#lifelines.utils.concordance_index) function to compute the concordance between a patient’s metastasis-free probability after 3 years and the patient’s ground truth event time. Following this definition, a perfect HCI for our model would be 1.0, as patients with e.g. a low value for metastasis-free probability should be patients with a short time-to-event.

### Implementation details

All the code needed for data pre-processing and running the models was written in Python 3.6. The networks were built and optimized with Tensorflow 2.2.0^40^ using the high-level library Keras^37^. Training and testing was carried out on three different graphical processing units: a NVIDIA P6000 with 24 GB, a NVIDIA P5000 with 16 GB and a NVIDIA Titan V with 12 GB of memory. The input shape of the 3D CT of $256 \times 256 \times 256$ and the batchsize of four were chosen so that the networks could fit also on the smallest graphical processing unit available. For the 2D-CNNs a batchsize of 32 was used. The weights and biases of the networks were optimized using the Adam algorithm^41^ with a constant learning rate of $3 \times 10^{-4}$ and $2 \times 10^{-4}$ for the 2D and 3D binary classification CNNs and of $6 \times 10^{-4}$ and $3 \times 10^{-4}$ for the 2D and 3D time-to-event CNNs. The respective loss functions used were binary cross-entropy and the custom loss function *surv_likelihood* by Gensheimer et al.^27^.

Prior to the augmentations, all images were isotropically re-sampled to a $1 \times 1 \times 1$ mm$^3$ grid. Specifically, the binary masks where re-sampled in 3D using in-house code for shape-based interpolation^42^. The full CTs where interpolated in 3D using linear interpolation. Moreover, all CT values were windowed to $-500$ and $+500$ Hounsfield Units and then re-scaled from $-1$ to $+1$ (standard image input). The linearly interpolated CTs were then masked with the shape-based interpolated binary masks to create the re-sampled masked CTs which were used as input to the networks. For the binary masking experiment, we set all values inside the GTV which were bigger than $-1$ to $+1$, i.e. we used re-sampled binary masks as input (see Supplementary Fig. S1).

To decrease over-fitting, a weight decay term of $1 \times 10^{-4}$ was used for all models. Additionally, for the 2D and 3D CNNs and the 2D and 3D CNNs+Clinical several augmentations were implemented by adapting a multithreaded augmentation pipeline which is freely available online^43^ (https://github.com/MIC-DKFZ/batchgenerators) to our workflow. This allowed us to use a slightly wider range of augmentations and especially to perform the augmentations online (during the training), in contrast to Diamant’s study. To be specific, we used random cropping to 128 pixels in each dimension with a central random shift of maximal 20% of the original $256 \times 256 \times 256$ image. We also used mirroring of the image with 50% probability, rotations of maximal 60 degrees and elastic deformations of the image of up to 25% of the size of the cropped image, both with a probability of 10% for the binary classification 3D-CNN, 20% for the time-to-event 3D-CNN and 50% for all 2D-CNNs. Finally, for all our models we also used dropout with a *dropout_rate* of 25%. For the CNNs all these hyper-parameters were found by performing a manual grid search meaning that we repeated the 3-fold CV on the Canadian benchmark data set until we were satisfied with the mean validation AUC or HCI which was achieved with given parameters. On the other hand, training for the ANN took much less, so after manually finding a range of parameters leading to good results we performed an automatic grid search over 16 different combinations of hyper-parameters (number of neurons in the hidden layer, weight decay, dropout probability, learning rate) to find the ones performing best on average for the 3-fold CV. For all CNN+Clinical models we manually explored some hyper-parameters around the best hyper-parameters for the CNNs and found that the ones leading to best average CV were the same as for the CNN.

For all our network architectures we took the 3 models achieving the best average CV performance and used them for testing without any further change. To get a single prediction out of three we applied the validation models on test data and then averaged the resulting three predictions to obtain a single model averaged prediction per patient. A schematic view of the workflow is shown in Fig. 1.

When doing binary classification the 3D-CNN and 3D-CNN+Clinical were trained for 500 epochs, using Keras callbacks to save the model’s weights when an improvement in the AUC was observed. One 3-fold CV took on average 11 h. The 2D-CNN and 2D-CNN+Clinical were trained for 500 epochs, one 3-fold CV taking on average 30 minutes. The ANN was trained for 5000 epochs, one 3-fold CV taking on average 10 minutes. When performing time-to-event analysis the 2D and 3D CNNs and CNNs+Clinical were trained for 300 epochs while the ANN for 3000, taking on average 8 h for the 3D-CNNs, 20 min for the 2D-CNNs and around 10 min for the ANN. For all the networks and the tasks, we used early stopping with a patience of 200 for all CNNs and 2000 for the ANN to shorten the overall training time.

### Statistical analysis

The performance of the different models on the different testing cohorts was investigated in a two-fold way: by looking at the discriminative power using the area under the receiver operating characteristic curve^44^ (AUC) for binary classification tasks or Harrell’s concordance index^32^ (HCI) for time-to-event tasks and by looking at patient stratification capability using a threshold optimised on the validation cohort. To find the threshold we first averaged over the risk of all validation patients who got a DM, then we averaged over the risk of all the validation patients which did not get a DM and finally we took the mean of these two thresholds for all three CV models and obtained a final model averaged threshold to be used for stratification of the testing sets.

Following the suggestions by^45,46^ we assessed significance of difference by using both estimation statistics and statistical tests. We applied estimation statistics^45^, i.e. we focused on the sizes of effects at level of data, by computing the median AUC or HCI with 83% confidence intervals from bootstrap re-sampling (consisting in generating many variants of a given dataset by repeatedly taking samples with replacement from the original set^32^). We used 83% confidence as it can be shown^47,48^ that two non-overlapping 83% confidence intervals mean that the two corresponding means/medians differ significantly with a significance level of 0.05. To be in line with literature^5,9,17^, we also split the testing patients into a high- and low-risk group and checked whether the stratification is significant by computing the p value for the log-rank test. We consider results with p values $< 0.05$ to be statistically significant.

## Supplementary Information

Supplementary Information 1.

## Data Availability

All the code needed to build the models and perform the analysis is freely available on GitLab: https://gitlab.physik.uni-muenchen.de/LDAP_ag-E2ERadiomics/dl_based_prognosis.

## Data availability

Three out of four cohorts used in this study can be found on The Cancer Imaging Archive (TCIA)^24^: Canadian benchmark dataset^23^: 10.7937/K9/TCIA.2017.8oje5q00. MAASTRO dataset^30^: 10.7937/tcia.2019.8kap372n. PMH dataset^31^: 10.7937/tcia.2019.8dho2gls. The CRO dataset is the result of a collaboration and can be obtained upon request.
