# Supplementary material for: Distant metastasis time to event analysis with CNNs in independent head and neck cancer cohorts
Source: Sci Rep. 2021 Mar 19;11:6418. doi: 10.1038/s41598-021-85671-y (PMC7979766; doi:10.1038/s41598-021-85671-y)
Supplement: Supplementary file 1 — Supplementary Information 1. [file 41598_2021_85671_MOESM1_ESM.pdf]

Supplementary Information: Distant metastasis time to event analysis with CNNs in independent head & neck cancer cohorts

Elia Lombardo[1,2], Christopher Kurz[1,2], Sebastian Marschner[1,3], Michele Avanzo[4], Vito Gagliardi[4], Giuseppe Fanetti[5], Giovanni Franchin[5], Joseph Stancanello[6], Stefanie Corradini[1], Maximilian Niyazi[1], Claus Belka[1,3], Katia Parodi[2], Marco Riboldi[2,+], and Guillaume Landry[1,2,+,*]

[1]Department of Radiation Oncology, University Hospital, LMU Munich, Munich, 81377, Germany
[2]Department of Medical Physics, Faculty of Physics, Ludwig-Maximilians-Universität München, Garching, 85748, Germany
[3]German Cancer Consortium (DKTK), Munich, 81377, Germany
[4]Medical Physics Department, Centro di Riferimento Oncologico di Aviano (CRO) IRCCS, Aviano, 33081, Italy
[5]Radiation Oncology Department, Centro di Riferimento Oncologico di Aviano (CRO) IRCCS, Aviano, 33081, Italy
[6]Guerbet SA, Villepinte, France
[*]guillaume.landry@med.uni-muenchen.de
[+]these authors contributed equally to this work

Supplementary results

Model	Image input	P-value MAASTRO	P-value PMH	P-value CRO
2D-CNN	standard	2e-3*	1e-3*	2e-3*
2D-CNN+Clinical	standard	3e-3*	< 1e-5*	4e-4*
3D-CNN	standard	5e-4*	< 1e-5*	< 1e-5*
3D-CNN+Clinical	standard	3e-3*	< 1e-5*	6e-3*
2D-CNN	binary	1e-1	2e-4*	1e-1
2D-CNN+Clinical	binary	4e-4*	< 1e-5*	5e-3*
3D-CNN	binary	3e-2*	< 1e-5*	6e-2
3D-CNN+Clinical	binary	4e-4*	< 1e-5*	1e-2*
ANN	-	4e-2*	< 1e-5*	5e-2*

Table S1. Comparison of p-values from the log-rank test applied on high- and low-risk splitted testing cohorts. Results are shown for all different time-to-event models and the two different image input scenarios. Note that p-values < 5e-2 are considered significant and marked with a *.

Model	Image input	3-fold CV	Test PMH-halved
2D-CNN+Clinical	standard	0.63; 0.72; 0.68	0.67 (0.60 - 0.75)
3D-CNN	standard	0.61; 0.74; 0.66	0.64 (0.56 - 0.72)
2D-CNN	binary	0.72; 0.73; 0.53	0.68 (0.61 - 0.74)
ANN	-	0.68; 0.59; 0.76	0.68 (0.61 - 0.75)

Table S2. Comparison of HCI values for CV on one half of PMH and testing of the other half of PMH. Results are shown for the two overall best time-to-event models (2D-CNN+Clinical and 3D-CNN), for the 2D-CNN which achieved the best HCI of 0.69 on the full PMH cohort when being trained on the Canadian benchmark dataset and for the ANN as baseline model.

Supplementary methods

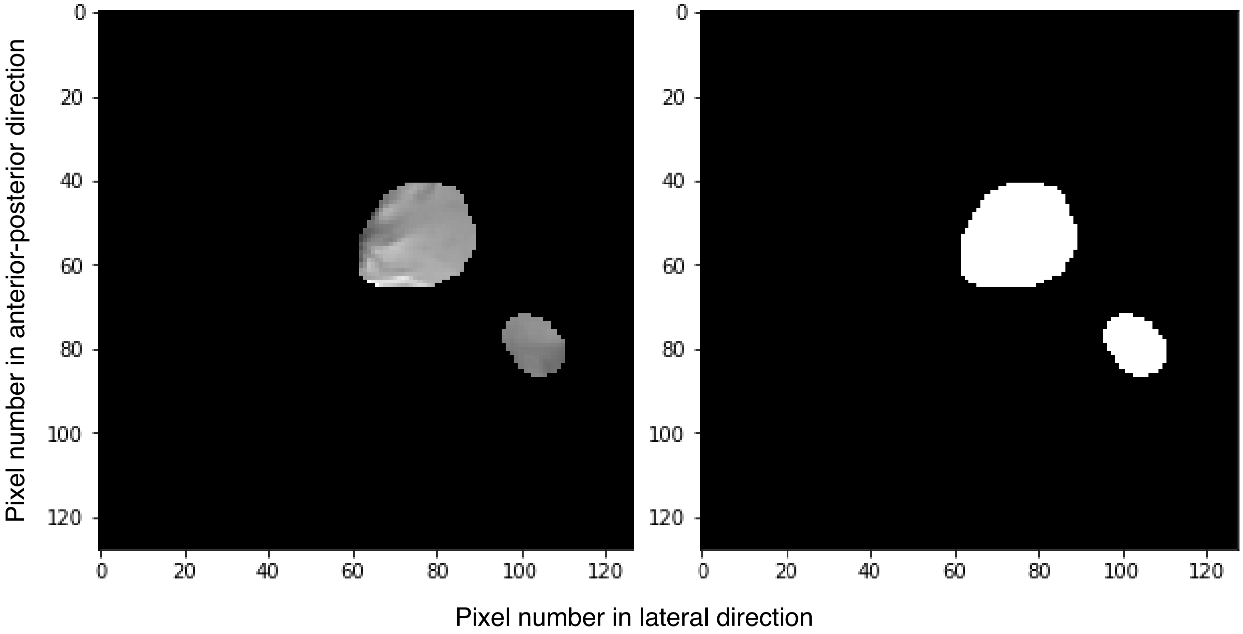

Figure S1. Standard image input vs. binary masking experiment for a selected CRO patient. (*Left*) Central axial 2D slice taken from the 3D input CT masked with the primary and lymph node GTVs. The values inside the GTV are re-scaled Hounsfield Units ranging from -1 to $+1$ while the black region outside is set to -1. (*Right*) Central axial 2D slice taken from 3D binary masked CT. All values inside the GTVs are set to $+1$ and everything outside is set to -1.
